# Detecting neonatal acute bilirubin encephalopathy based on T1-weighted MRI images and learning-based approaches

**DOI:** 10.1186/s12880-021-00634-z

**Published:** 2021-06-22

**Authors:** Miao Wu, Xiaoxia Shen, Can Lai, Weihao Zheng, Yingqun Li, Zhongli Shangguan, Chuanbo Yan, Tingting Liu, Dan Wu

**Affiliations:** 1 Key Laboratory for Biomedical Engineering of Ministry of Education, College of Biomedical Engineering and Instrumental Science, Zhejiang University, Hangzhou, 310027 China; 2 Department of Neonatal Intensive Care Unit, Children’s Hospital, Zhejiang University School of Medicine, Hangzhou, 310051 China; 3 Department of Radiology, Children’s Hospital, Zhejiang University School of Medicine, Hangzhou, 310052 China; 4 State Key Laboratory of Pathogenesis, Prevention and Treatment of High Incidence Diseases in Central Asia, College of Medical Engineering and Technology, Xinjiang Medical University, Urumqi, 830011 China

**Keywords:** Acute bilirubin encephalopathy, Hyperbilirubinemia, Normalized T1-weighted intensities, Deep convolutional neural networks, ResNet18, Classification, Diagnosis

## Abstract

**Background:**

Neonatal hyperbilirubinemia is a common clinical condition that requires medical attention in newborns, which may develop into acute bilirubin encephalopathy with a significant risk of long-term neurological deficits. The current clinical challenge lies in the separation of acute bilirubin encephalopathy and non-acute bilirubin encephalopathy neonates both with hyperbilirubinemia condition since both of them demonstrated similar T1 hyperintensity and lead to difficulties in clinical diagnosis based on the conventional radiological reading. This study aims to investigate the utility of T1-weighted MRI images for differentiating acute bilirubin encephalopathy and non-acute bilirubin encephalopathy neonates with hyperbilirubinemia.

**Methods:**

3 diagnostic approaches, including a visual inspection, a semi-quantitative method based on normalized the T1-weighted intensities of the globus pallidus and subthalamic nuclei, and a deep learning method with ResNet18 framework were applied to classify 47 acute bilirubin encephalopathy neonates and 32 non-acute bilirubin encephalopathy neonates with hyperbilirubinemia based on T1-weighted images. Chi-squared test and t-test were used to test the significant difference of clinical features between the 2 groups.

**Results:**

The visual inspection got a poor diagnostic accuracy of 53.58 ± 5.71% indicating the difficulty of the challenge in real clinical practice. However, the semi-quantitative approach and ResNet18 achieved a classification accuracy of 62.11 ± 8.03% and 72.15%, respectively, which outperformed visual inspection significantly.

**Conclusion:**

Our study indicates that it is not sufficient to only use T1-weighted MRI images to detect neonates with acute bilirubin encephalopathy. Other more MRI multimodal images combined with T1-weighted MRI images are expected to use to improve the accuracy in future work. However, this study demonstrates that the semi-quantitative measurement based on T1-weighted MRI images is a simple and compromised way to discriminate acute bilirubin encephalopathy and non-acute bilirubin encephalopathy neonates with hyperbilirubinemia, which may be helpful in improving the current manual diagnosis.

## Background

Neonatal jaundice, which develops in about 60% of term and 80% of preterm babies during their first week of life, is one of the most common conditions that require medical attention in newborns [1, 2]. It is mainly caused by the inability of the newborn’s immature liver to process the excessive bilirubin that was produced by the accelerated breakdown of red blood cells at this age[3, 4]. Although most jaundice is benign, 8–9% of infants might develop severe hyperbilirubinemia (HB), with approximately 4% affected after 72 h [5]. In even more serious, due to the lack of appropriate diagnoses and delayed treatments, severe HB patients may develop acute bilirubin encephalopathy (ABE) in response to the entry of bilirubin toxicity into the basal ganglia and various brain nuclei. And a long-term outcome of ABE could be kernicterus which is a permanent disabling neurologic condition classically characterized by the movement disorders of dystonia and choreoathetosis, hearing loss caused by auditory neuropathy spectrum disorders, and oculomotor pareses [6]. A recent study indicated that ABE accounts for 3.4% of neonatal admissions with 21.4% of those infants in severe conditions and at least 15% of them died [7]. Therefore, early diagnosis of neonates with a high risk of ABE and timely taking effective intervening measures are very important for pediatricians to minimize the mortality or prevent them from kernicterus.

The total serum bilirubin (TSB) measurement is a traditional and most widely used method for screening and diagnosing HB in neonates, but it needs a blood draw which is invasive and carries a risk of infection and anemia [8]. Meanwhile, as it is not a direct measurement of actual bilirubin level in the brain, TSB alone could not accurately predict the occurrence of ABE [9, 10]. Magnetic resonance imaging (MRI), as a non-radiation and non-invasive imaging technique, is widely used in the ABE diagnosis in newborns [11]. Many MRI studies found that newborns following ABE in the first days to weeks showed an increased signal on T1-weighted images (T1WI) at globus pallidus (GP) and subthalamic nucleus (STN) in most cases, while T2-weighted imaging of these regions was often unremarkable or shows subtle T2-hyperintensity[12–16]. Although the T1 hyperintensity in GP provides an efficient marker for diagnosing ABE neonates, it remains challenging to further separate ABE and non-ABE HB patients since both groups may demonstrate elevated T1 signals. On the other hand, not all ABE patients develop abnormalities in their T1WI at the time. A study from Mao [17] reported that 20 of 36 HB neonates have symmetric hyperintense GP on T1WI; and among these 20 HB neonates, 15 of 20 were ABE neonates. Another study from Wang et al. [14] reported 19 of the 24 ABE patients in their study were observed T1 hyperintensity in the bilateral GP while other 5 of 24 were not; and among these 19 patients, 10 of 19 had high T1 intensities in the STN while others had not. Coskun et al. [12] investigated the GP involvement in 13 neonates with ABE, and 8 of them demonstrated bilateral, symmetric increased signal intensity in GP on T1WI, while others did not. These studies based on manually radiological reading were subjective and prone to bias since various degree of T1 singal might be involved and there was not a standard for how high the T1 intensity can be a hyperintensity, which may result in different results for different observers. Therefore, improving the sensitivity and specificity of identifying ABE from HB patients based on T1WI alone remains challenging in conventional radiological reading.

In recent years, computer-aided diagnosis (CAD) technology was wildly used to improve the radiologist’s performance [18–20]. One of the important CAD methods named deep convolutional neural networks (CNN) was applied in our study, which demonstrated remarkable ability in diagnosing a variety of neurological diseases [21–25]. In this study, we compared 3 approaches to differentiate the ABE and non-ABE neonates from a cohort of HB babies based on routine clinical 2D multislice T1WI, including (1) radiological reading, (2) semi-quantitative analysis with normalized T1 intensities, (3) CNN-based classification with ResNet18 [26]. We systematically evaluated the diagnostic accuracy of 3 approaches in a retrospective study of 79 HB patients including 47 ABE and 32 non-ABE neonates. To the best of our knowledge, this is the first work to use semi-quantitative assessment and CNN in ABE diagnosis.

## Methods

### Study subjects

All procedures performed in this study involving human participants were following the ethical standards of the institutional and national research committee and with the 1964 Helsinki declaration and its later amendments or comparable ethical standards. Informed consent was obtained from all individual participants included in the study. All the MR images were collected retrospectively from routine clinical scans at the Children’s Hospital of Zhejiang University School of Medicine between the years of 2009 and 2018. A total of 79 HB patients (ABE/non-ABE = 47/32, male/female = 52/27), who had MRI examinations at chronological age from 1 to 18 days during their hospitalization, were selected. The diagnostic criteria for ABE positive cases met either of the following clinical diagnosis criteria, including (1) sever hyperbilirubinemia; (2) at least one of the ABE-related clinical symptoms with bilirubin-induced neurologic dysfunction (BIND) score ≥ 1 point, where 1, 2, or 3 points corresponding to mild, moderate, or severe symptoms based on the severity of the crying pattern, behavioral and mental status, and muscle tone (a total of 9 points). Overall BIND score of 1–3 points, 4–6 points, 7–9 points represented subtle signs of mild ABE, moderate ABE, and advanced ABE, respectively. At last, 47 ABE and 32 non-ABE (HB) cases were confirmed by 2 experienced pediatric radiologists. (S.XX and C.L.).

### MRI Acquisition

T1WI was acquired on a 3.0 T Achieva scanner (Philips Healthcare, Best, the Netherlands) using a 2D multislice T1-weighted fast field-echo sequence in the axial direction with the following parameters: echo time of 2.14 ms, repetition time of 200 ms, flip angle of 80°, field-of-view of 330 × 330 mm, resolution of 0.45 mm, and 18 slices with a thickness of 4.5 mm. All slices were visually examined by the radiologists with high image quality and none of them were excluded.

### Visual inspection of the MR images

We invited three pediatric radiologists, including a senior radiologist with 12 years of experience (C.L.) and 2 fellows (L.Y. and Z. S.) with 7 years of experience, to independently review the MR images. Since there are currently no radiological standards for diagnosing ABE, the raters were first trained by reviewing all the MR images with true labels (ABE or non-ABE) and the corresponding clinical information, such as age, sex, TSB, etc. One week after the training, they were asked to review the images again without the labels nor the clinical information to make a diagnosis decision based on T1WI only. The images were shuffled with re-assigned identification numbers at the training and testing sessions.

### Semi-quantitative diagnosis with normalized T1 Intensity

**Fig. 1:**
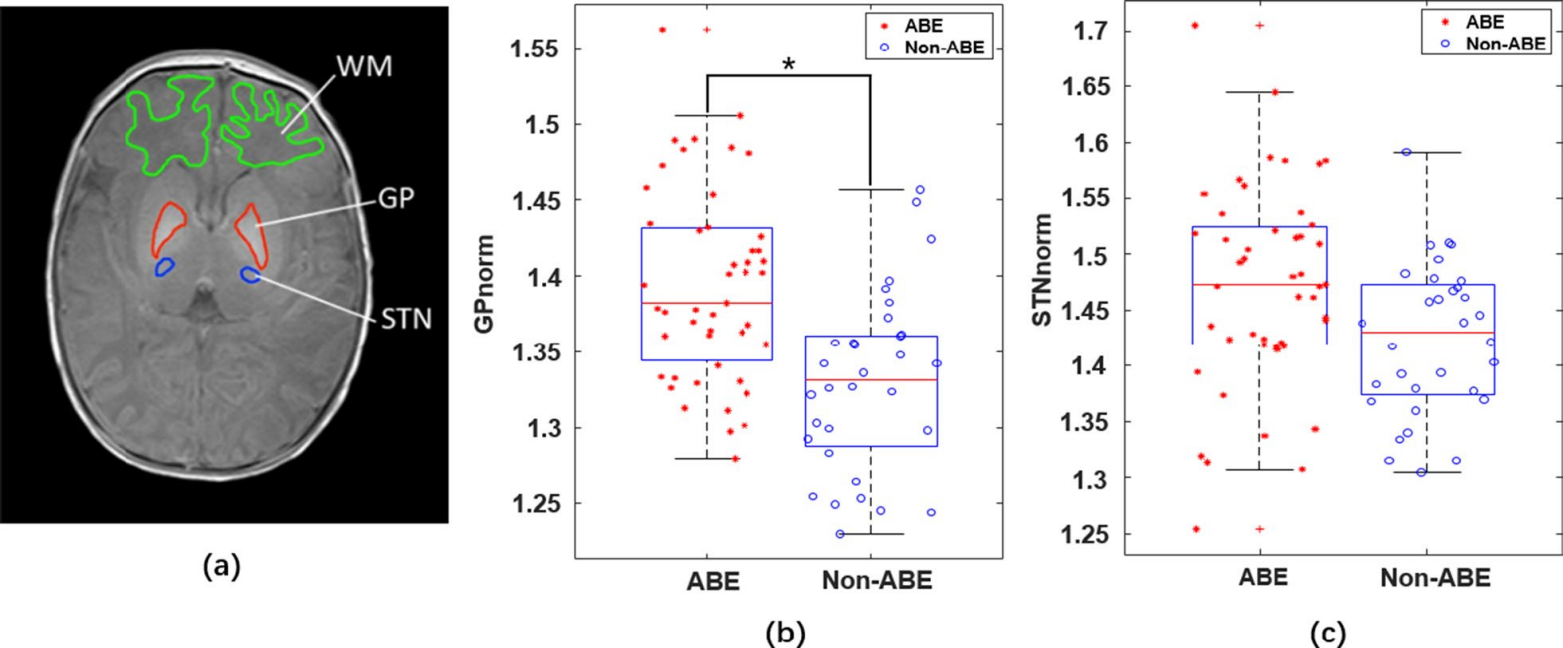
ROI of the image and distribution map of patients’ GP _norm_ and STN _norm_. **a** ROI definitions. Green: white matter, red: globus pallidus, blue: subthalamic nucleus. GP_norm_ (**b**) and STN_norm_ (**c**) in the ABE and non-ABE HB patients

We chose a slice from T1WI of each HB neonate covering the largest area of GP and STN for analysis. Region of interest (ROI) was manually delineated on the selected slice, including the anterior subcortical white matter (WM), GP, and STN, as shown in Fig. 1a. WM was used as the reference region to normalize the T1-signals in GP or STN, becasue there are no known T1-signal changes in WM between the ABE and non-ABE patients. The normalized T1 intensities of GP and STN were defined as

1 $${\text{GP}}_{{{\text{norm}}}} = \frac{{\overline{{GP}} }}{{\overline{{WM}} }}$$

2 $${\text{STN}}_{{{\text{norm}}}} = \frac{{\overline{{STN}} }}{{\overline{{WM}} }}$$

where $\overline{{GP}}$, $\overline{{STN}}$, and $\overline{{WM}}$ denote the averaged T1WI intensities in the GP, STN, and WM ROIs, respectively. We then applied the Youden Index [27–29] in the software of IBM SPSS Statistics 21 to determine the optimal cut-off threshold of GP_norm_ and STN_norm_ for separating ABE and non-ABE patients, respectively.

### Deep learning framework

In this section, we describe the deep learning procedure for classification work. 2 or 3 continuous T1WI slices covered the GP from each patient were selected as inputs of the CNN. A total of 190 slices were collected, including 95 randomly selected slices from ABE patients (approximately 2 continuous slices per patient) and 95 slices from non-ABE patients (approximately 3 continuous slices per patient). All selected images were normalized between 0 and 1 with a min–max normalization algorithm.

As our classifier was performed based on a pre-trained CNN in Matlab 2019a (https://www.mathworks.com), which requires 3 channels image input with a size of 224 × 224x3 pixels. Consequently, the normalized image was resized into 224 × 224 pixels and then replicated to 3 channels to form a 224 × 224x3 image which served as an input of the network. The dataset was randomly split into a training dataset and testing dataset with 80% and 20% split ratio, respectively. Then, fivefold cross-validation was followed to estimate the model’s performance.

**Fig. 2:**
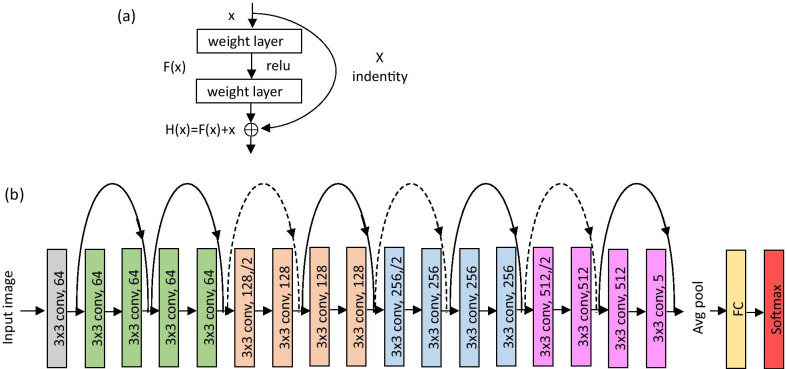
**a** The basic residual block. **b** The ResNet18 architecture. The numbers in each convolutional layers denote the number of filters

We employed a CNN of Resnet18 [26] which consists of 18 residual blocks, where each residual block is defined as:

3 $$y = {\text{F}}\left( {x,\left\{ {Wi} \right\}} \right) + x$$

where $x$ and $y$ were the input and output, and ${\text{F}}\left( {x,\left\{ {Wi} \right\}} \right)$ represented the residual mapping to be learned. ResNet18 applied residual learning to every few stacked layers. A residual block was different from conventional CNN architecture in the existence of a shortcut connection between the input and output, serving as an identity projection for alleviating the vanishing gradient issue in deep networks[26], as shown in Fig. 2a. The mapping function H(x) = F(x) + x was realized as a residual shortcut connection in a feedforward neural network and performs element-wise addition.

The ResNet18 architecture was shown in Fig. 2b, containing 18 learnable layers. The convolutional layers used 3 × 3 filters, and the downsampling was performed for every 4 layers after the input layer by convolutional layers with a stride of 2. Note the number of filters get doubled as a downsample took place. At the end of ResNet18, an average-pooling was applied followed by a fully-connected layer and a softmax layer. Residual shortcut connections denoted as the curves in Fig. 2b were added throughout the network. The solid curves were used when input and output had the same dimensions; while the dotted curves were used when the dimension increased, where the shortcut performed identity mapping with zeros padding for the increased dimension with a stride of 2.

Since the size of our dataset was limited, we applied the transfer learning approach for our classification model [30]. The weights of ResNet18 were initialized by pre-training on the ImageNet [31] and then fine-tuned with our datasets. Data augmentation was also applied on the training datasets to enhance our model’s performance, which included image rotation with a random angle in the range of -30° to 30°, image vertical flipping with 50% probability, images zooming by a random scale within the range of 0.9 to 1.1, image horizontal and vertical translation with random distance in the range of −30 to 30 pixels. The learning rate was initialized to 0.0003, MaxEpoch = 6, Stochastic Gradient Descent Momentum based solver is used with a minibatch size of 10 images for training.

To investigate which brain areas influence our classification results most, we applied the class activation mapping (CAM) to each testing subject. CAM is a technique used to get visual interpretations of the regional contributions to the predications of CNN [32].

The model was implemented under the environment of Matlab 2019a on a computer having specifications of 16 GB RAM and Inter® Core™ i7-8700, CPU@ 3.20 GHz, GPU NVIDIA Geforce GT 730.

### Statistical analysis

The group differences in the sex distribution among groups were evaluated using the chi-squared test, while other clinical features were evaluated by t-test.

The group differences in GP_norm_ and STN_norm_ between the ABE and non-ABE patients were accessed by using analysis of covariance (ANOVA) with age, sex, gestational age at birth, and PMA as covariates.

To evaluate the classification performances of different methods, serval performance metrics were applied in this study, including sensitivity, specificity, precision, F1-score, and the area under the curve (AUC) of the Receiver Operating Characteristic (ROC) curves [33, 34]. χ^2^-test was applied to determine the significant differences in the classification accuracy by different methods.

All statistical analysis was performed using IBM SPSS Statistics 21 (https://www.ibm.com/products/spss-statistics).

## Results

The demographic and clinical characteristics of the HB patients were listed in Table 1, including the patient’s sex, age, weight, gestational age, TSB, and albumin. The results were shown in Table 1.

**Table 1:** The demographic and clinical characteristics of the HB patients used in this study

Clinical features	ABE positive (n = 47)	ABE negative (HB) (n = 32)	*p* value
Sex (male)	29(61.70%)	23(71.88%)	0.349
Age (days)	9.83 ± 3.05	12.15 ± 5.28	0.032
Weight (kg)	3.21 ± 0.48	3.36 ± 0.43	0.162
Gestational age (weeks)	38.47 ± 1.58	38.38 ± 1.47	0.792
TSB (μmol/L)	369.11 ± 114.78	326.13 ± 79.20	0.070
Albumin (g/L)	38.34 ± 2.98	38.45 ± 3.21	0.873

**Fig. 3:**
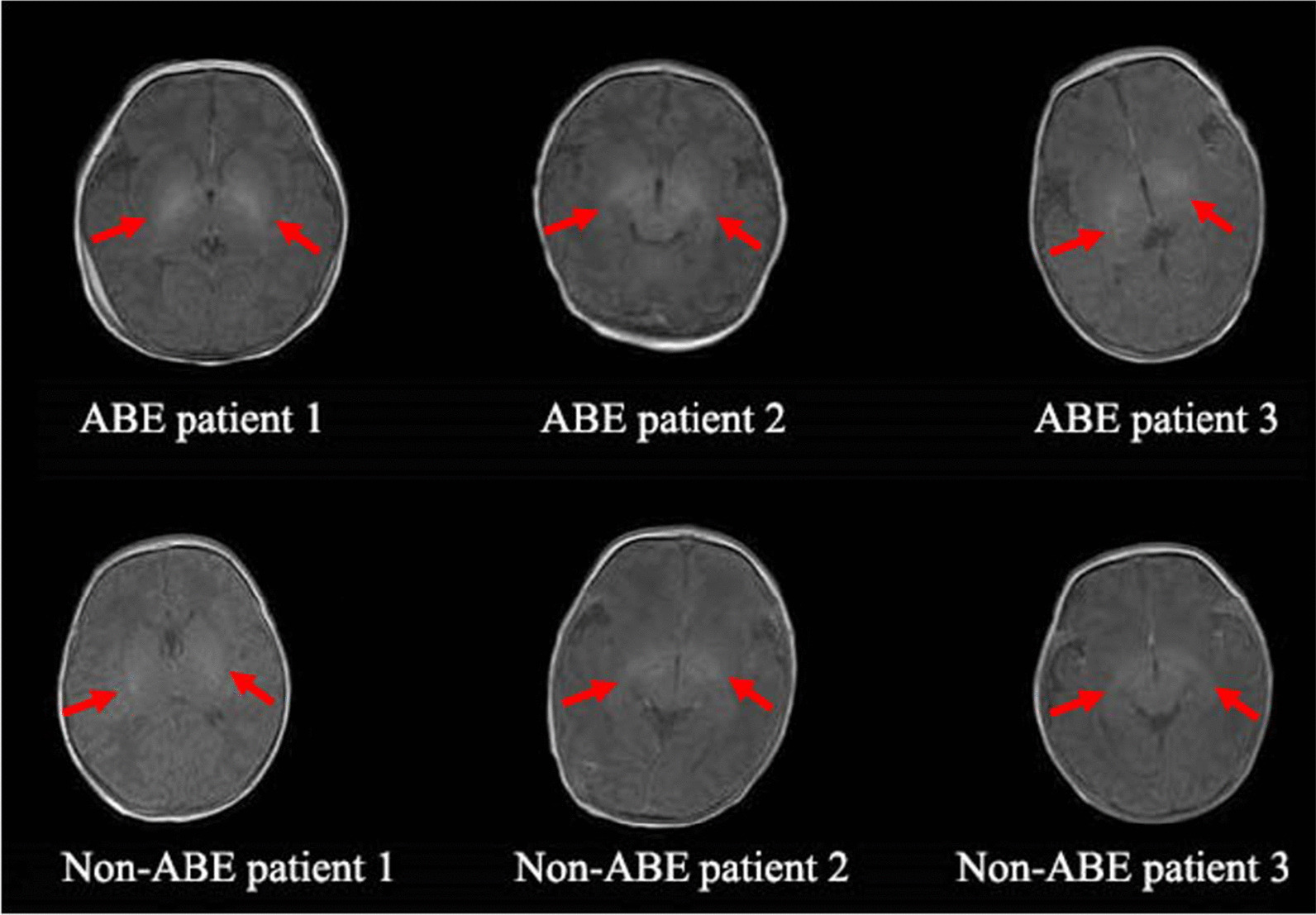
Representative T1WI from three ABE and three non-ABE neonates who were diagnosed as HB. The arrows pointed to the bilateral areas of the globus pallidus

Figure 3 showed several representative T1WI from ABE and non-ABE patients who were diagnosed with HB. The two groups exhibited similar image features with hyperintensity in the GP and large individual variations were observed.

### Results of visual inspection of the MR images

**Fig. 4:**
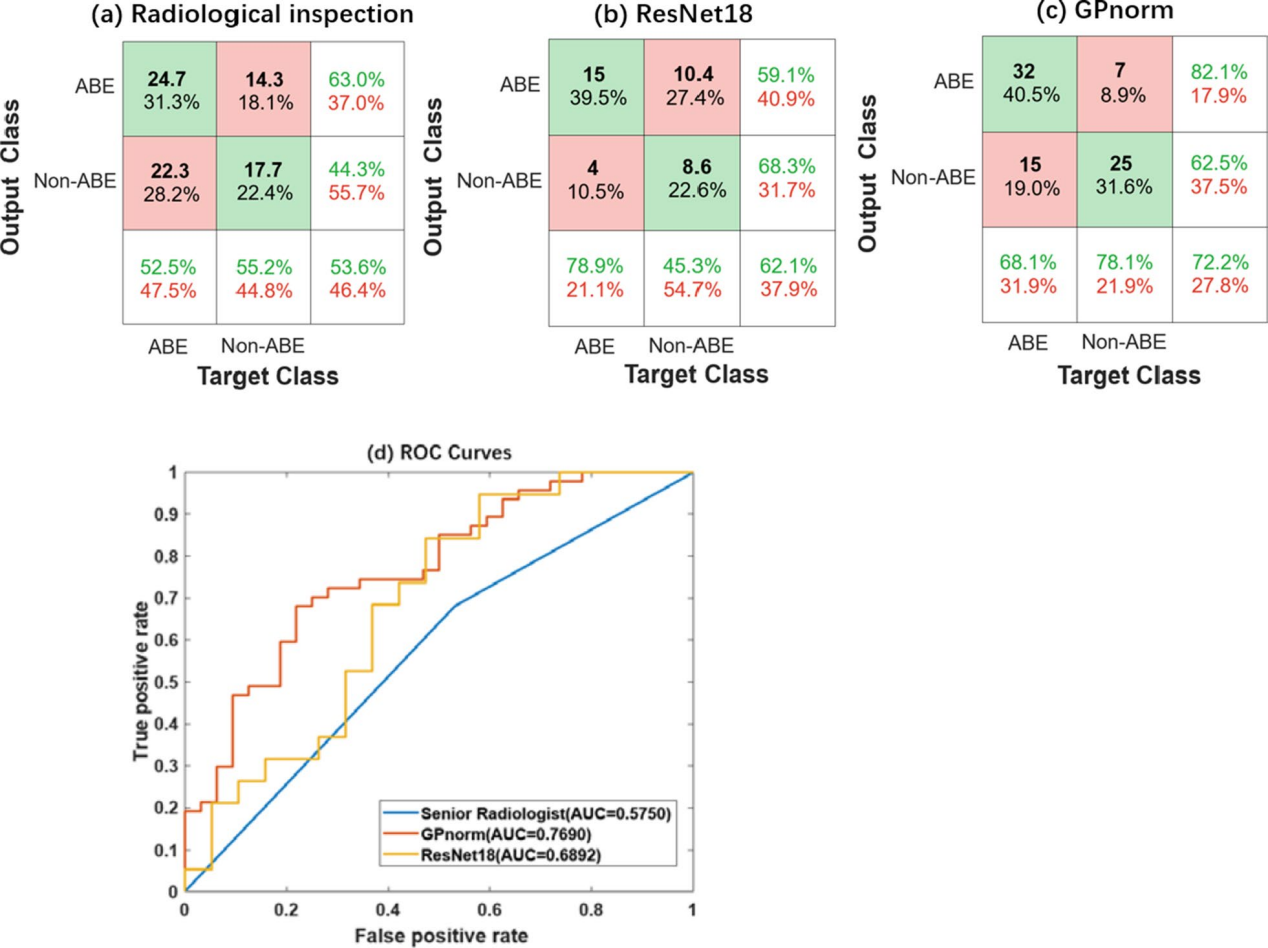
Confusion matrices and ROC curves of classification results of three different methods. **a** Confusion matrix based on the radiological inspection. **b** Confusion matrix based on ResNet18. **c** Confusion matrix based on semi-quantitative measurement of GP_norm_. **d** ROC curves for three different methods. The corresponding AUC values were denoted in the lower right corner

We recorded the 3 experienced radiologists’ visual diagnosis results, and their average diagnostic results were 52.48%,55.21%,62.95%,0.5679,53.58%,0.5387 for sensitivity, specificity, precision, F1-score, accuracy, AUC, respectively, shown in Table 2. The results indicated the difficulty in separating the 2 groups by conventional radiological reading in real clinical practice. The overall Fleiss’ kappa coefficient for intraobserver reliability is 0.5082 (*p* < 0.05), indicating the agreement of 3 radiologists was moderate and not accidental. Furthermore, χ^2^-test also indicated that there was no statistically significant difference in the results between 3 radiologists’(*p* > 0.05). The ROC curve generated based on the diagnosis results of the senior radiologist, which was the best among the three raters, was shown in Fig. 4d (blue curve).

**Table 2:** The classification performance of visual inspection, GP_norm_, and ResNet18 in separating ABE from non-ABE HB patients, as evaluated by sensitivity, specificity, precision, F1-score, Accuracy, AUC

Methods	Sensitivity	Specificity	Precision	F1-Score	Accuracy	AUC
Visual inspection	52.48 ± 13.58%	55.21 ± 7.86%	62.95 ± 3.58%	56.79 ± 8.90%	53.58 ± 5.71%	53.87 ± 4.11%
GP_norm_	68.10%	**78.10%**	**82.05%**	**74.42%**	**72.15%**	**76.90%**
ResNet18	**78.95 ± 17.85%**	45.26 ± 19.19%	59.58 ± 7.09%	67.11 ± 8.28%	62.11 ± 8.03%	68.92 ± 11.06%

The maximum value of performance metrics for each method was marked in bold

### Results of Semi-quantitative diagnosis with normalized T1 intensity

The distribution of normalized T1 intensity in GP and STN were shown in Fig. 1b and Fig. 1c for the ABE and non-ABE patient groups. The t-test indicated that a significant difference between the ABE and non-ABE in the GP_norm_ (1.39 ± 0.06 and 1.33 ± 0.06, *p* < 0.05), but no significant difference in the STN_norm_ (1.47 ± 0.09 and 1.42 ± 0.07, *p* > 0.05). The ROC curve based on GP_norm_ was shown in Fig. 4d (orange curve). The AUC was 0.769 for GP_norm_, and 0.678 for STN_norm_, respectively. The optimal cut-off thresholds based on the Youden Index were 1.3621 and 1.5118 for GP_norm_ and STN_norm_, respectively.

### Results of ResNet18

The diagnostic performance of ResNet18 as evaluated by five-cross validation was: 78.95 ± 17.85%, 45.26 ± 19.19%, 59.58 ± 7.09%, 67.11 ± 8.28%, 62.11 ± 8.03%, 68.92 ± 11.06% for sensitivity, specificity, precision, F1-score, accuracy, AUC, respectively, which were listed in Table 2.

**Fig. 5:**
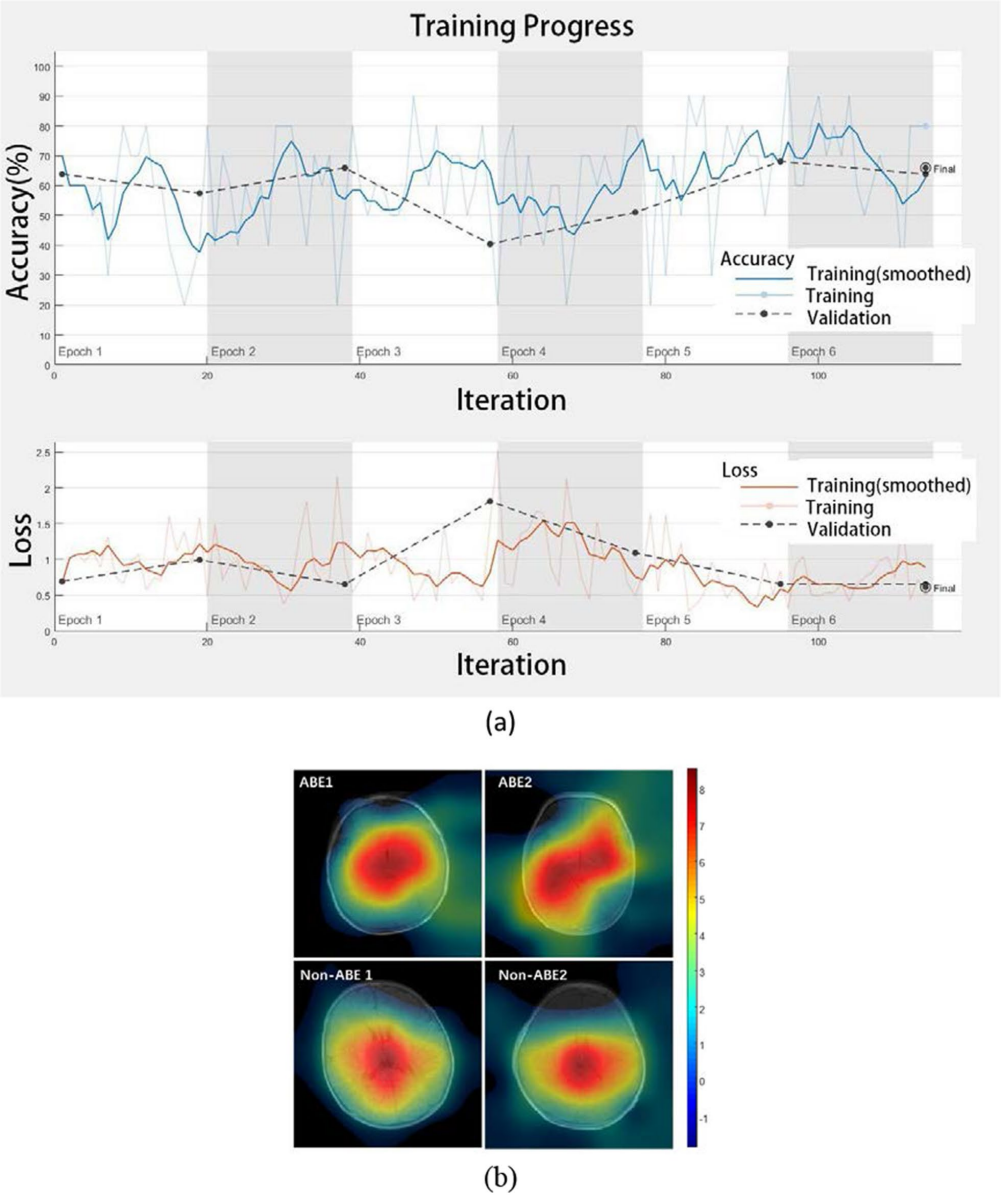
**a** A training progress of ResNet18 in fivefold cross-validation: the accuracy and loss history. **b** Class activation map of resnet18 for 4 exemplary test samples. The colormap showed the contribution of the voxels in the network in predicting results and the red region contribute most

Figure 5b demonstrated four examples of the CAM on the testing samples that were correctly predicted by the network. The red regions in the CAM represented the brain regions that contributed most in predicting the results, and they were mostly located in the center of the brain covering the areas of bilateral GP and STN.

### Comparison of three methods

The classification performances of different methods were compared in Table 2. The semi-quantitative method based on GP_norm_ showed superior performance compared to the other two approaches except for the sensitivity measure. Figure 4a-c was the confusion matrix of classification results of three different methods. Figure 4d showed the ROC curves based on the three methods for direct comparison. χ^2^-test indicated that the accuracy of GP_norm_ marker was significantly higher than that of visual inspection (*p* = 0.014), but no difference was found between the results of visual inspection and ResNet18 (*p* = 0.234) or between results of ResNet18 and GP_norm_ (*p* = 0.153).

## Discussion

Currently, the radiological finding of ABE is T1 hyperintensity in the areas of GP and STN since they were affected by the bilirubin [14, 35]. A previous study [36] indicated that the relatively high resting neuronal activity in the GP and STN are postulated to make them more vulnerable to oxidative stresses from mitochondrial toxins, such as bilirubin, or genetic mitochondrial disorders. Such damages to the GP and STN of the ABE infants can be often observed on their T1WI, resulting in T1 hyperintensity in various degrees [14, 35]. However, the sensitivity and specificity of this radiological feature are only moderate since only T1WI is studied without any other complementary and useful information [12] [14] [17]. A future study including multi-modal MRI and clinical information of the patients is expected to improve the diagnostic performance.

Our study aimed to investigate the utility of T1WI for the diagnosis of ABE in neonates. 3 different diagnostic methods are performed. As shown in Table 2, the accuracy and AUC from low to high are visual inspection (53.58 ± 5.71%, 0.5387 ± 4.11%), ResNet18 (62.11 ± 8.03%, 68.92 ± 11.06%), and semi-quantitative diagnostic method with GP_norm_ (72.15%, 0.7690). The study demonstrated that the performance of conventional radiological reading based on T1WI for diagnosing ABE is unsatisfactory. Advanced analytical approaches with deep learning and semi-quantitative measurement outperformed the conventional radiological reading, and therefore, offering the opportunity to improve the diagnostic accuracy of the ABE in clinical practice.

Although MRI has been increasingly applied to investigate the neuropathology induced by ABE in the neonatal clinical practice, the efficiency and accuracy of the conventional radiological reading strategy solely based on visual inspection of GP and STN on T1WI were hardly satisfactory. This is because the T1 intensity of GP and STN in ABE demonstrated a high extent of heterogeneity and the level of T1 hyperintensity may be confounded by the normal development of GP as well as myelination in the adjacent posterior limb of the internal capsule. There is no clear boundary between ABE and normal neonates, nor to mention the distinction between ABE and non-ABE HB neonates, e.g., some non-ABE cases also showed slight T1 hyperintensity in GP and STN (Fig. 3). Besides, the diagnosis of routine visual inspection is qualitative and subjective, e.g., we observed a relatively high inter-rater variability among the three raters (Fleiss’ kappa coefficient = 0.5082, *p* < 0.05).

**Fig. 6:**
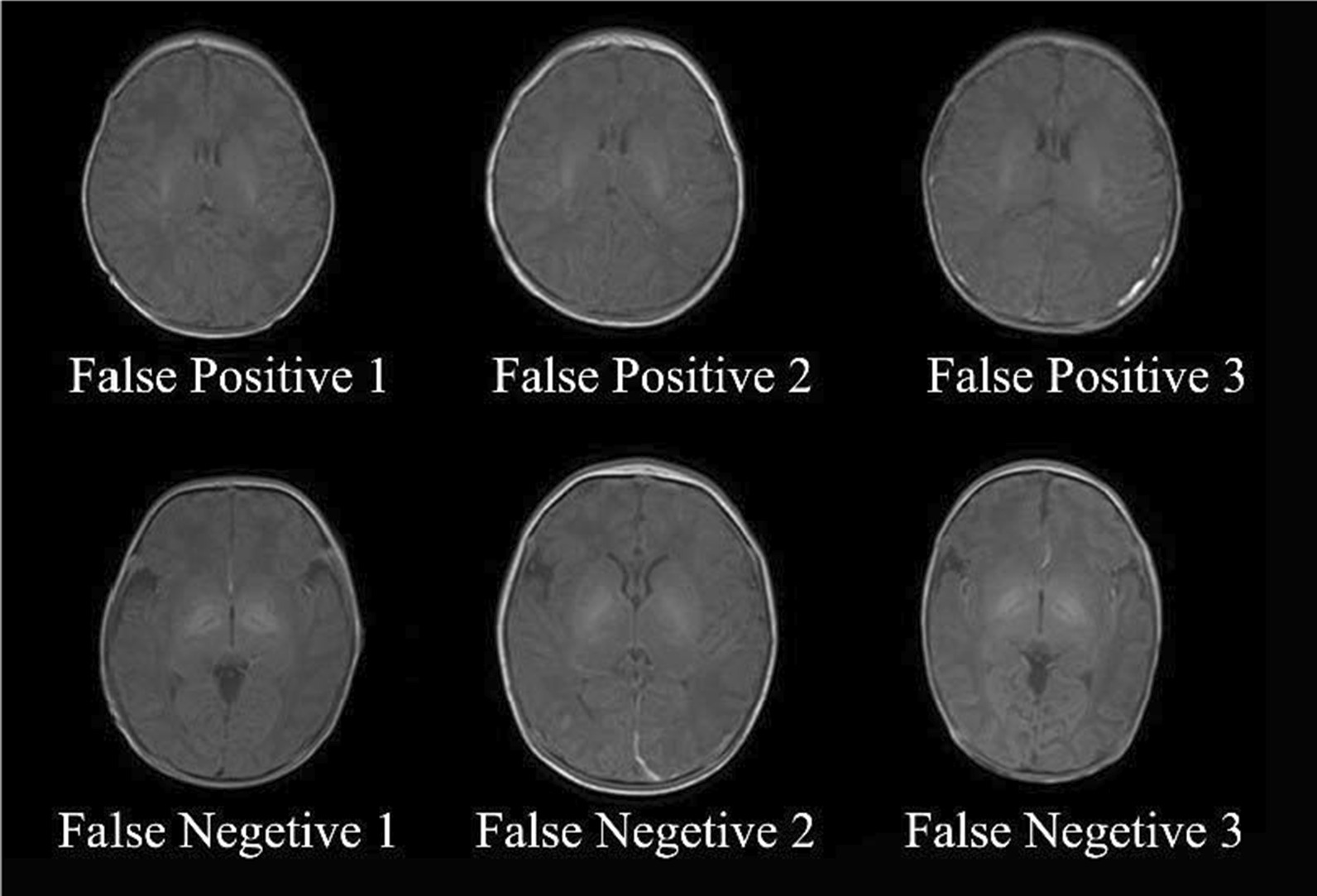
Examples of false-positive cases (non-ABE HB patients who were misclassified as ABE) and false-negative cases (ABE patients who were misclassified as non-ABE HB) by the ResNet18 network

As the deep learning technology has been wildly used in medical image analysis, we applied the deep learning model ResNet18 to T1WI-based diagnosis of ABE. The CAM map in Fig. 5 indicated the center regions of the brain covering bilateral GP and STN played a critical role in the classification task. This was consistent with our prior knowledge that most ABE patients followed an increased T1-signals in GP and STN [12–17]. However, the improvement in diagnostic accuracy was only moderate (from 53.58% for visual inspection to 62.11% for ResNet). It because some image samples applied in our study have not prominent or even inverse manifestation between ABE and non-ABE. The misclassified samples by ResNet18 shown in Fig. 6 indicated that some non-ABE images have a prominent T1 hyperintensity in GP, whereas some ABE images did not show visual abnormalities. It is a common phenomenon in clinical practice as the hyperintensity of GP on T1WI is only a clinical manifestation of ABE that is likely to occur but not necessarily[12, 14], which indicates that ABE diagnosis based only on T1WI is not sufficient in the clinical practice. Thus, additional clinical information is needed to support the diagnosis of ABE, such as TSB, Albumin, etc.[37]. Also, we found that during the training process, the overall tendency of training accuracy did not increase much during the training process, which indicated that ResNet18 cannot perfectly differentiate the samples that did not have instinct features. We noticed that in Fig. 5a validation loss increased after 40 iterations and the training loss kept decreasing, which indicated overfitting took place. This, in turn, means that the complexity of our model was greater compared to the limited training samples. Moreover, the generalizability of our model is unknown, which is a known caveat of CNN for small sample size data [38]. Additional studies, ideally covering a large number of cases from multiple centers, are needed to further improve the diagnosis of neonatal ABE.

To circumvent the issues related to small sample size, model complexity, and generalizability, we took a simple and model-free approach using the semi-quantitative based on normalized T1 intensity in GP and STN regions. We found a statistical difference in GP_norm_ between ABE and non-ABE and there was no distinct line yet; meanwhile, no statistical difference was found in STN_norm_. Therefore, an optimal threshold of 1.3621 was determined to separate ABE patients from HB neonates based on GP_norm_, which achieved significantly improved diagnostic performance compared to the visual inspection. This semi-quantitative diagnostic pipeline would is expected other datasets given the minimal requirement on preprocessing, computational power, and training data. We deem that GP can be observed at T1WI and its T1-intensity may have a subtle variation when different MRI equipment was applied. However, the value of GP_norm_ would not be changed as it is a normalized value. Nevertheless, the results of all three experiments demonstrated that it is not enough to make an accurate diagnosis only based on the T1WI alone. Therefore a study combining the information of T1WI and other MRI modalities, i.e. T2-weighted, diffusion-weighted MRI, etc. is essential in the future work for a more accurate diagnostic result.

## Conclusion

The current study investigates the utility of T1WI in diagnosing ABE conditions through three analytical approaches. The semi-quantitative diagnostic method provided the highest performance followed by ResNet18, which both outperformed the conventional visual inspection strategy. In particular, the semi-quantitative GP_norm_ achieved the highest accuracy of 72.15% and AUC of 76.90%. Our work showed advanced analytical approaches to make the best use of conventional T1WI which would assist the diagnosis of ABE in real clinical practice.

## Data Availability

The datasets generated and/or analyzed during the current study are not publicly available as they contain identifiable and personal information but are available from the corresponding author on reasonable request.

## Abbreviations

ABE: Acute bilirubin encephalopathy
HB: Hyperbilirubinemia
TSB: Total serum bilirubin
MRI: Magnetic resonance imaging
T1WI: T1-weighted images
GP: Globus pallidus
STN: Subthalamic nucleus
WM: White matter
CAD: Computer-aided diagnosis
CNN: Convolutional neural networks
BIND: Bilirubin-induced neurologic dysfunction
CAM: Class activation mapping
ROI: Region of interest
AUC: Area under the curve
ROC: Receiver Operating Characteristic
